# Welcoming new Editors to Disease Models & Mechanisms

**DOI:** 10.1242/dmm.049120

**Published:** 2021-05-28

**Authors:** E. Elizabeth Patton, Kirsty Hooper

**Affiliations:** 1 MRC Human Genetics Unit and Cancer Research UK Edinburgh Centre, MRC Institute of Genetics and Molecular Medicine, The University of Edinburgh, Western General Hospital, Crewe Road South, Edinburgh EH4 2XU, UK; 2 The Company of Biologists, Bidder Building, Station Road, Histon, Cambridge CB24 9LF, UK

Disease Models & Mechanisms (DMM) publishes disease-focused high-quality research that is freely accessible to everyone (Patton, 2021). Our new Editor-in-Chief, Elizabeth Patton, was appointed in January 2021. Part of Liz's vision for DMM is to strengthen the journal's presence in the field of translational biology and to focus on the importance of disease *mechanisms*, just as much as *models* (Vyas, 2020; Patton, 2021). Liz identified four thematic challenges that are imperative for advancing disease biology: mechanisms of disease, innovative technologies, disease progression through time and therapy (Patton, 2021). To address these challenges, DMM aims to promote multidisciplinary collaborations between basic biologists, clinical scientists, computational biologists and bioengineers.

To implement this vision, DMM has appointed new Editors Sally Dunwoodie (Victor Chang Cardiac Research Institute, Australia), Monkol Lek (Yale School of Medicine, USA) and Rickie Patani (Francis Crick Institute, London, UK). Each new Editor has strong influence in their respective field, and a unique perspective on how basic science underpins our understanding of disease aetiology, and progress in diagnosis and treatment.

## Sally Dunwoodie

Every year, 4.9 million serious birth defects occur globally. Prof. Sally Dunwoodie studies mammalian embryogenesis to determine the environmental and genetic factors that contribute to these birth defects. Her major focus is congenital heart disease; she is the founder of Australia's largest genome sequencing initiative for this disease. However, her discoveries have also included vertebral defects, as Sally has identified several NOTCH-associated genes that cause defects by disrupting somite patterning. Sally is a world-leading researcher in developmental genetics and inherited disease, with her research leading to enhanced genetic diagnostic testing worldwide. This exemplifies tangible clinical outcomes; Sally will be invaluable to help generate a clear sense of clinical relevance in DMM.

Sally completed her PhD on the genetic control of muscle development at the Children's Medical Research Institute and the University of Sydney, then further trained at the National Institute for Medical Research in London with Rosa Beddington. Sally is now a Professor in the Faculty of Medicine at the University of New South Wales and bases her lab in the Victor Chang Cardiac Research Institute. A particularly ground-breaking moment in Sally's lab was the discovery in 2017 that deficiency in nicotinamide adenine dinucleotide, an essential coenzyme in metabolism of all cells, causes recurrent miscarriage and birth defects in humans and mice. This was prevented by supplementation with niacin (vitamin B3) during pregnancy in mice. Sally's research has had significant clinical impact; therefore, she will have exceptional insight into the translational potential of articles submitted to DMM.

**Figure:**
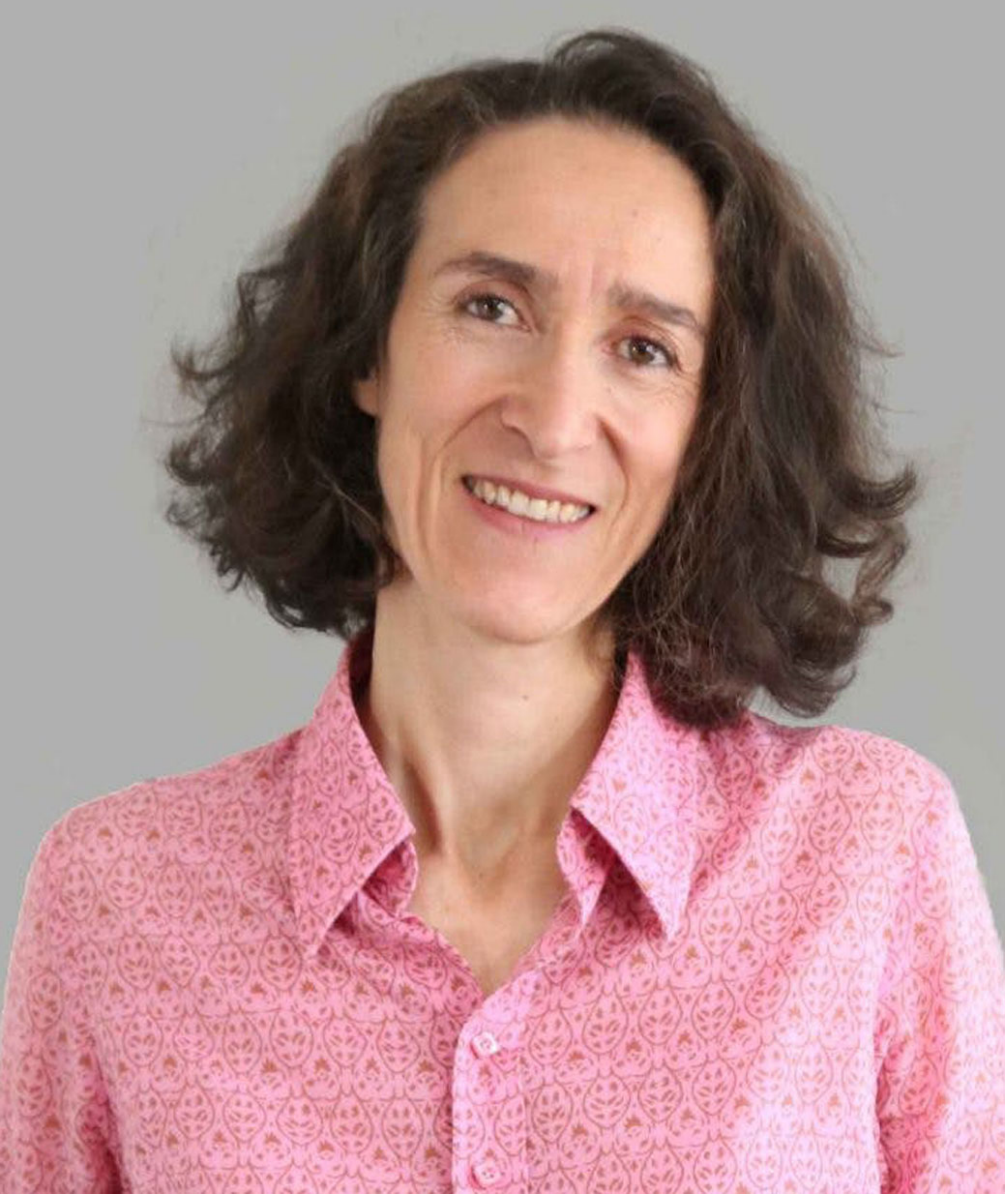
Sally Dunwoodie

## Monkol Lek

Currently, only 50% of rare genetic disease patients receive a diagnosis. The lack of understanding of rare diseases, such as muscular dystrophy, also means that there is no cure and limited treatment options. Through engagement with the DMM community, we identified rare diseases as an area of future focus (Vyas, 2020). Dr Monkol Lek's lab focuses on improving diagnosis rates for rare disease, and also translating genetic diagnosis to therapies, such as gene editing technologies.

The use of large datasets was another area identified by our community for expansion within DMM (Vyas, 2020). Monkol's pioneering work on large-scale genetic studies further emphasizes his importance to the DMM Editorial team; he also has a unique perspective on rare genetic diseases. He originally studied computer engineering, but while working at IBM received a clinical diagnosis of muscular dystrophy. In pursuit of a clearer understanding of the genetics behind his condition, Monkol retrained in biology and completed a PhD in Kathryn North's lab at the University of Sydney, studying the genetics of muscle strength and performance. In the final year of his PhD, he received a genetic diagnosis for his disease, which further motivated him to help other patients receive their genetic diagnosis. Monkol then joined Daniel MacArthur's lab at Harvard Medical School, where he was part of a team that built a resource to help clinical genetics labs identify variants associated with rare diseases. The Exome Aggregation Consortium (ExAC) project has been accessed over 5 million times worldwide and is cited approximately three times a day. He then led several other large-scale projects using computational methods, which have resulted in novel disease gene discovery. Monkol became Assistant Professor and started his own lab at Yale in 2018, where he focuses his research on patients in underserved populations, such as those in East Asia, whose genetic mutations are not as well characterised as those of Europeans. Monkol's dedication to promoting essential research like this will strengthen DMM's vision to enable meaningful clinical impact.

**Figure:**
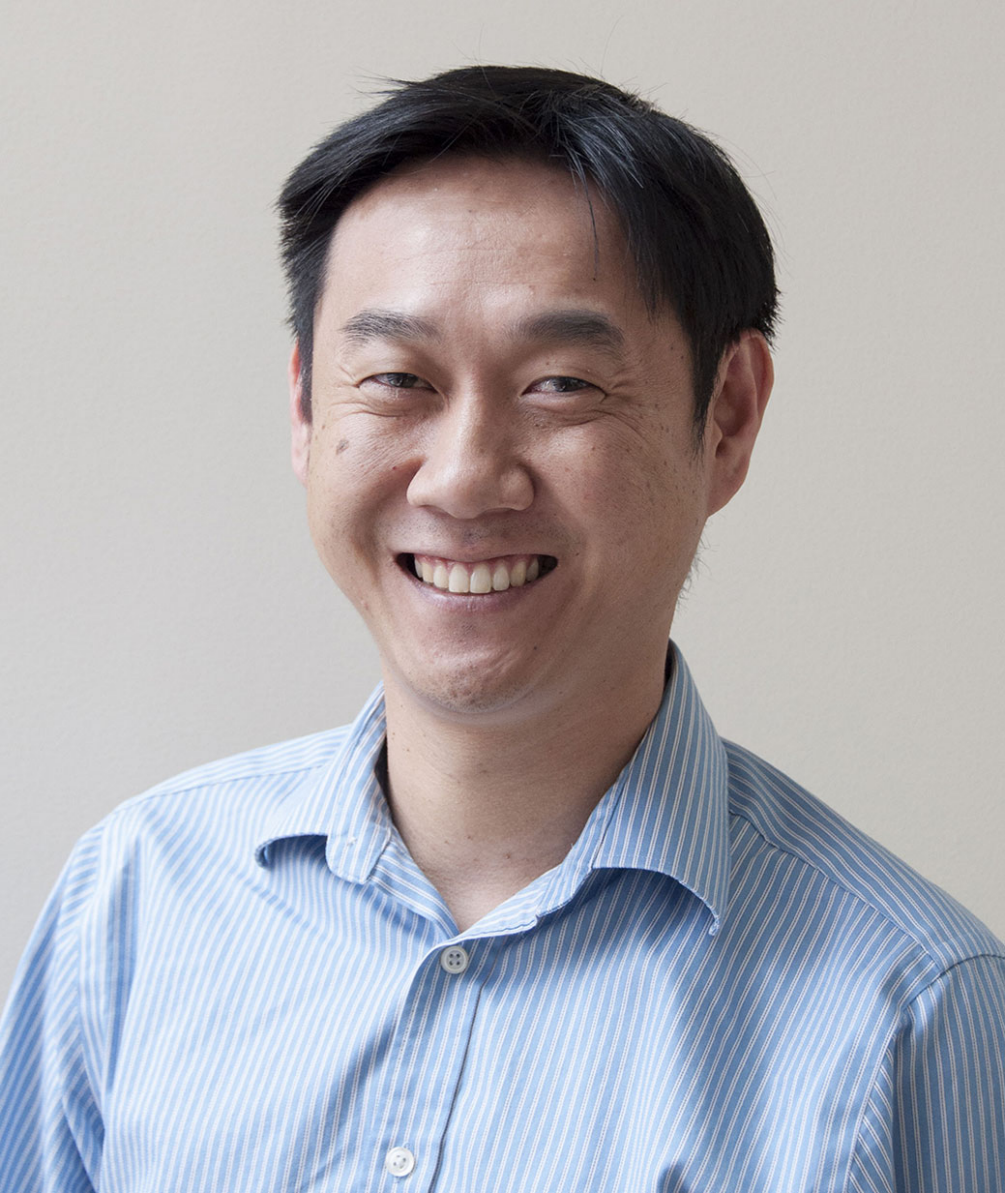
Monkol Lek

## Rickie Patani

Amyotrophic lateral sclerosis (ALS), the most common form of motor neuron disease, is a rapidly progressive neurodegenerative disease, which leads to muscle weakness and paralysis, and is ultimately fatal. This condition remains incurable due to a paucity in understanding its aetiology. Prof. Rickie Patani's work has the goal of inhibiting and/or reversing the characteristic motor neuron damage in this disease. To achieve this, Rickie's lab has developed techniques that differentiate ALS-patient-derived stem cells into nerve and glial cells to allow delineation of disease mechanism. In her recent Editorial, Liz highlighted DMM's desire to engage scientists utilising human cell and tissue models in their translational research (Patton, 2021). With Rickie bringing his expertise in human induced pluripotent stem cell models of neurodegeneration, as well as RNA metabolism and cellular autonomy, to DMM's Editorial team, we are better equipped at implementing this goal.

As Rickie is a physician scientist, he can also offer a clinically focused perspective on prospective DMM articles. He completed his PhD at the University of Cambridge in human stem cell neurobiology in 2011, and pursued his passion for neurology by gaining higher clinical training in neurology and starting his research group at University College London in 2013. In 2017, Rickie was appointed Honorary Consultant Neurologist at the National Hospital for Neurology and Neurosurgery and moved his lab to the Francis Crick Institute. There, he progressed to Professor in 2019 and was awarded the Graham Bull Prize and Goulstonian Lectureship from the Royal College of Physicians in 2020. Rickie's expertise in neurology and stem cell biology, combined with his dedication to improving clinical outcomes for his patients, makes him a perfect addition to the DMM Editor team and will enable us to forge strong connections with clinical science.

**Figure:**
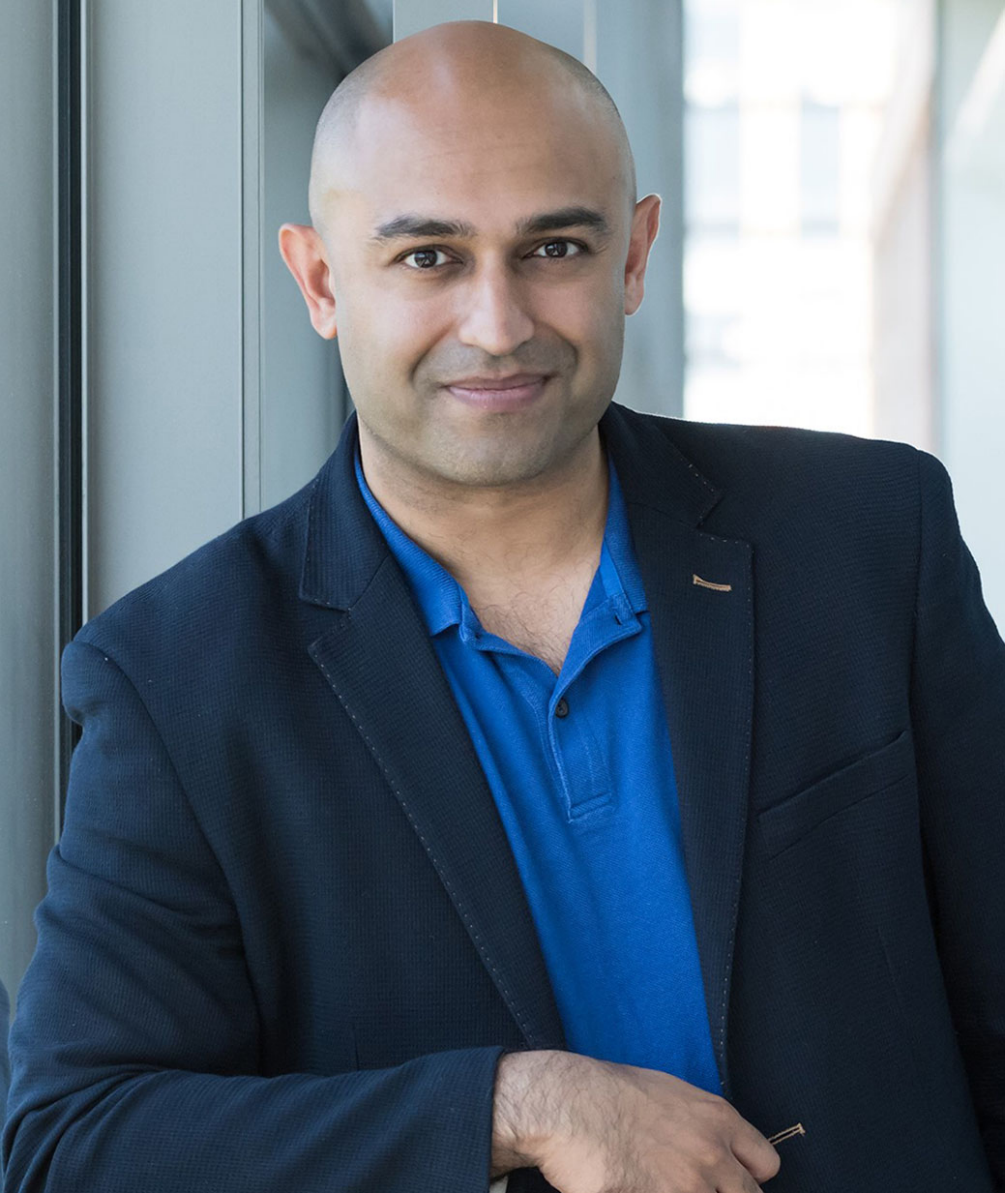
Rickie Patani
